# Human Resources in Primary Health-Care Institutions before and after the New Health-Care Reform in China from 2003 to 2019: An Interrupted Time Series Analysis

**DOI:** 10.3390/ijerph19106042

**Published:** 2022-05-16

**Authors:** Chenyuan Qin, Min Liu, Xin Guo, Jue Liu

**Affiliations:** 1 Department of Epidemiology and Biostatistics, School of Public Health, Peking University, No. 38, Xueyuan Road, Haidian District, Beijing 100191, China; qincy@bjmu.edu.cn (C.Q.); liumin@bjmu.edu.cn (M.L.); 2 Department of Institutional Reform, National Health Commission of the People’s Republic of China, No. 1, Xizhimen Wainan Road, Xicheng District, Beijing 100044, China; 3 Institute for Global Health and Development, Peking University, Beijing 100871, China

**Keywords:** primary health-care institutions, health workforce, health-care reform, interrupted time series analysis

## Abstract

At the end of March 2009, the Chinese government put forward the new health-care reform with its long-term goal to achieve universal access to basic medical and health services. Primary health-care institutions and adequate health human resources are the basic and specific guarantee to achieve the above goals. We aimed to explore the differences in the trends of human resources before and after the new health-care reform from 2003 to 2019, based on *Chinese Health Statistics Yearbook*, an using interrupted time series analysis. The number of primary health workers serving for every 1000 people in China gradually rose from 1.98 in 2003 to 3.07 in 2020, with an immediate sharp rise from 2008 to 2009. Subsequently, the number of primary health workers increased at a significant speed, but it still failed to meet people’s basic medical needs (3.5/1000). There was an increase of 51,301 primary health staff in 2010, and 6007 more primary health workers per year were added after the reform compared to the basic increasing number before 2009, but both were not statistically significant (*p* > 0.05). The annual increased number of health workers in township health centers was 2.63 times greater than that before the reform, with a total annual increase of 34,192 people (*p* < 0.05). The growth trend changed the most in western China (*p* < 0.001), while the human resources of primary health-care institutions in the eastern region were relatively richer than those in the central and western regions. Numerous registered nurses and pharmacists have been devoted to the construction of the primary health-care service since 2010, while the increase in the number of doctors per year was not statistically significant (*p* = 0.066). In total, the primary health-care human resource in China is constantly expanding, but the primary health-care needs are still not fully met, especially for doctors, and the problem of unbalanced allocation urgently needs to be solved. Reasonably formulating regional policies to strengthen the construction of primary health personnel and expand the accessibility of primary health services is the top priority in the development of primary medical and health undertakings.

## 1. Introduction

The World Health Organization (WHO) is clear in its definition of universal health coverage (UHC): all people can access the basic health services they need without financial hardship caused by payment [1]. Well-functioning health systems, financing systems, available medicines and technologies, and trained health-care personnel are all decisive factors influencing health equity [1]. UHC is driving the global health agenda, with countries at all stages embracing it as an important goal for health development [2]. Primary health-care institutions are the specific and necessary foundation in China to gradually provide universal coverage of basic healthcare services [3], which mainly include community health service centers (stations), township health centers, village clinics, etc. At the end of March 2009, the Chinese government put forward the *Opinions on deepening the reform of the medical and health system* (known as “the new health-care reform”), with the overall goal to establish and improve the basic medical and health system covering urban and rural residents and to provide safe, effective, convenient, and affordable health services to the masses [4]. The Chinese government hoped that by 2020 every resident could have access to basic medical and health services to meet the multilevel medical needs of the people and further improve their health. Under the guidance of the reform outline, the government has made great efforts to develop the rural medical and health service system and improve the new urban medical and health service system based on community health service [4]. Obviously, adequate health human resources are the basic guarantee to achieve the above goals, which could directly affect the diagnosis and treatment effect and service level of primary health-care institutions [5].

Primary medical and health personnel are an important part of a country’s health human resources, which include medical practitioners, registered nurses, pharmacists, etc. [5]. Unfortunately, *Chinese Health Statistical Yearbook 2021* reported that the number of staff in primary health-care institutions was only 4,339,745 in 2020, and each staff member was supposed to serve more than 350 people [5]. Compared with patients’ demand for medical services, the quantity of medical and health services provided by primary health-care institutions was still in short supply [5,6]. Medical services provided by primary health-care institutions mainly include the diagnosis and treatment of common and frequently occurring diseases, the rehabilitation and nursing of some diseases, and the acceptance of referred patients. The disease spectrum and aging reality in China determine that the absolute number and proportion of the treatment volume in primary health-care institutions should not decline [4]. From 2009 to 2019, the proportion of people visiting primary health-care institutions among the three major medical institutions dropped from 61.85% to 51.97% [7]. Furthermore, the number of consultations showed absolute declines in 2015 and 2018, and the proportion of inpatients dropped from 31.14% in 2009 to 16.17% in 2019 [7].

However, most of the previous studies mainly focused on the overall evaluation of the effectiveness and shortcomings of China’s new health-care reform and did not notice the changing level of human resources in primary health-care institutions before and after the reform, based on national data [2,4,6,8]. Therefore, we performed an interrupted time series analysis and planned to explore the impact of the new health-care reform on human resources in primary health-care institutions using the publicly available data from 2003 to 2019 in the health statistics yearbook. With the continuous progress of the reform, we wanted to understand whether the existing needs of the Chinese people for basic health services can be met, whether the development of primary health-care workers was balanced among different regions, and the possible directions of policy adjustment for the development of primary health-care human resources and the health equity of society.

## 2. Methods

### 2.1. Data Resources

The human resources data of primary health-care institutions from 2003 to 2019 were obtained from the *Chinese Health Statistical Yearbook* (2003–2011), *Chinese Health and Family Planning Statistical Yearbook* (2012–2016), and *Chinese Health Statistical Yearbook* (2017–2019). All data were collected from the public database, and no informed consent was required.

### 2.2. Data Collection and Research Indicators

We extracted and sorted out the human resources data of Chinese primary health-care institutions in 31 provinces from 2003 to 2019 and classified them by region if available, and 11, 12, and 8 provinces were incorporated into the eastern region, the western region, and the central region, respectively.

Community health service centers (stations), township health centers, village clinics, and other clinics constitute the main structure of the primary medical and health system. Due to missing data for the number of personnel in community health service centers (stations) by region from 2003 to 2006, we only obtained regional data from 2007 to 2019, that is, the corresponding table only showed the average change rate from 2007 to 2019, 2007 to 2009, and 2010 to 2019 in this category. The personnel data of township health centers in Beijing and Shanghai were deleted to maintain consistency because of the loss and confusion of classification. Health workers in village clinics did not include registered nurses. The staffs of primary health-care institutions were classified into doctors (medical practitioners or medical assistant physicians), registered nurses, pharmacists, technicians, and other job positions. Additionally, the overall human resources in primary health-care institutions were obtained directly from the statistical yearbooks, other than the sum of those three institutions.

### 2.3. Data Analysis

The mean, standard deviation, and the average annual growth rate were used to describe the number and the trends of staff of different primary health-care institutions and job positions among each time period. We used the interrupted time series (ITS) and piecewise linear regression model to analyze the variation trends of human resources before and after the new health-care reform [9].

Considering that the policies for new health-care reform were released at the end of March 2009 [4], we set 2003–2009 and 2010–2019 as the times before and after the new health-care reform, respectively. The piecewise linear regression model was set as: Y = β_0_ + β1 × T + β2 × reform + β3 × (T − T0) × reform + ε. Y represented the number of staff members; T was the number of years since the beginning of the study (set as “1” in 2003); Reform represented the intervention state of the observation point, and we set “0” during the status before the implementation; T_0_ meant the start time of the intervention, and (T − T_0_) represented the number of years since the intervention implementation; ε meant the random error. β_0_ was the baseline level of the population at the beginning of our study; β_1_ was interpreted as the slope of the number of personnel before the reform; β_2_ was the level change following the intervention; and β_3_ indicated the slope change after the implementation of the new health-care reform, which meant an extra rise compared to β_1_. Moreover, (β_1_ + β_3_) represented the whole slope of the number of personnel after the intervention. Whether the level change and slope change were statistically significant could be explained by the test results of the coefficient hypothesis.

In this study, Durbin–Watson statistics were used to evaluate whether the dependent variables were autocorrelated, and the generalized least squares method (Paris–Winsten method) was used for the subsequent correction [10]. Our statistical analysis was performed using R4.0.3, and a *p* value less than 0.05 was considered statistically significant.

## 3. Results

### 3.1. Descriptive Analysis of Human Resources in Different Primary Health-Care Institutions and Job Positions

**Figure 1:**
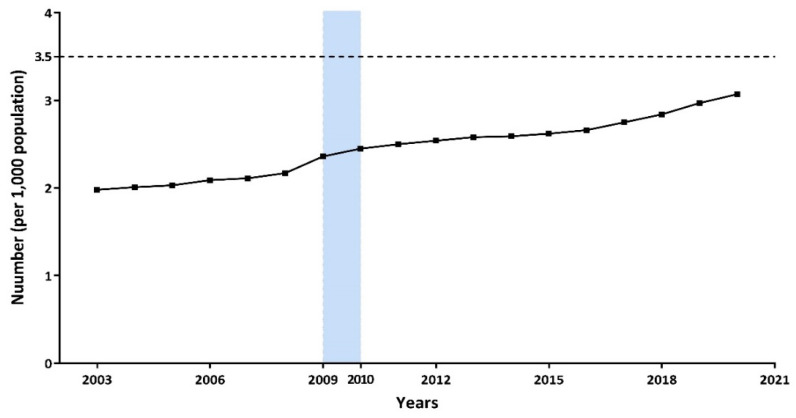
Number of primary health workers per 1000 population from 2003 to 2020.

The number of primary health workers per 1000 people in China gradually rose from 1.98 in 2003 to 3.07 in 2020, but this still failed to meet people’s basic medical needs (Figure 1). We found that before 2009 the number of primary health workers (per 1000 population) only increased at an extremely low rate, and every two health workers were supposed to provide basic health services for nearly 1000 people. However, the new health-care reform was introduced at the end of March 2009, and there was an immediate sharp rise from 2008 to 2009. Subsequently, the number of primary health workers increased at a significant speed after the reform, and it exceeded three primary health workers per 1000 population in 2020. On average, approximately 2.77 million people were employed in the primary health-care institutions before the new health-care reform (2003–2009), and this number quickly increased by 30% to 3.638 million after the reform (Table 1). The human resources of primary health-care institutions in the eastern region were relatively richer than those in the central and western regions. Community health service centers (stations) had the lowest number of staff compared to township health centers and village clinics, which were 0.16 million, 1.04 million, and 1.05 million, respectively, before the reform but then increased to 0.50 million, 1.28 million, and 1.32 million, respectively. Among them, the average number of staff in community health service centers (stations) after the new reform was 3.24 times that before the new reform. In terms of different job positions, the numbers of doctors, registered nurses, pharmacists, and technicians before the new intervention were 0.83 million, 0.33 million, 0.12 millon, and 0.06 million, while after the new health-care reform, the number of doctors, registered nurses, and technicians increased by 35%, 100%, and 57%, respectively.

According to the Outline of the National Medical and Health Service System Plan (2015–2020), China should have had at least 3.5 primary health workers for every 1000 permanent residents by 2020.

From 2003 to 2009, the number of staff in primary health-care institutions grew annually at a rate of 3.52%, which was slightly faster than that following the intervention (2.67%) (Table 1). In terms of each classification, the average growth rate of primary health workers in western China was faster than that of the other two institutions. Human resources in community health service centers (stations) maintained an obvious increasing trend by region, but the growth rate slowed down after the new health-care reform. However, the average growth rate crept up faster in township health centers (2.56%) than before (1.27%). The eastern region had the fastest growth rate of human resources. Excluding registered nurses, the total number of health personnel in village clinics showed the fastest growth rate (6.40%) in the central region before the new health-care reform. From 2003 to 2009, the annual growth rate of the number of village health staff in the eastern region was 3.82%, while it has declined by −0.20% each year since 2010. For the different job positions compared with the circumstance before the reform, the total number of doctors, registered nurses, and pharmacists increased markedly, with annual growth rates of 4.71%, 8.35%, and 2.16% from 2010 to 2010. However, the average annual growth rate of the number of technicians dropped from 6.86% to 4.00% in the context of the new health-care reform.

### 3.2. Interrupted Time Series Analysis of Human Resources in Primary Health-Care Institutions from 2003 to 2019

**Figure 2:**
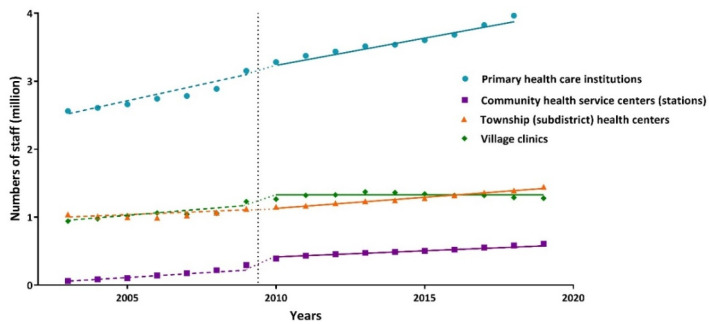
Interrupted time series analysis of human resources in various primary health-care institutions in China from 2003 to 2019.

Our results showed an overall upward trend of human resources in primary health-care institutions before the new health-care reform, with an increasing number of 91,581 (*p* < 0.001) per year. There was an immediate increase of 51,301 primary health staff from 2009 to 2010, and 6007 more primary health workers per year were added after the reform compared to the basic increasing number before 2010, but both increases were not statistically significant (*p* > 0.05) (Table 2, Figure 2).

From the perspective of different primary health-care institutions, the number of staff in community health service centers (stations), township health centers, and village clinics increased steadily before the new health-care reform, among which the number of staff in community health service centers (stations) and village clinics both increased by about 37,000 per year on average. However, the annual increased number of health workers in township health centers and village clinics after the reform in western China was not statistically significant (*p* > 0.05). Instantaneous changes after the reform showed that the number of people in community health service centers (stations), township health centers, and village clinics increased by approximately 96,700, 24,800, 131,500, respectively. However, the instantaneous level change of health workers in township health centers after the reform had no statistical significance (*p* = 0.375). With an average decline of 15,032 a year compared to the basic increasing number before 2010, the personnel number of community health service centers (stations) slowed down after the new health-care reform. The annual increased number of health workers in township health centers was 2.63 times than that before the reform, with a total annual increase of 34,192 people (*p* < 0.05). The growth trend changed the most in western China (*p* < 0.001). However, there was no significant difference in the extra change of the growth trend in eastern China (*p* = 0.076) and central China (*p* = 0.771) after the reform (Table 2 and Figure 2).

**Figure 3:**
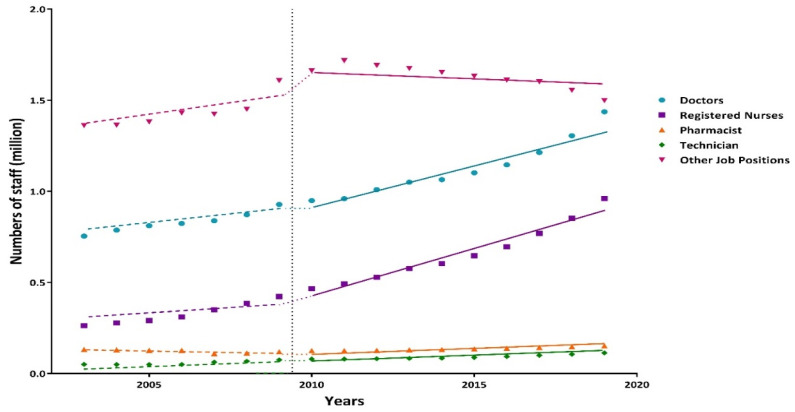
Interrupted time series analysis of human resources in different job positions of Chinese primary health-care institutions from 2003 to 2019.

As for human resources in different job positions of Chinese primary health-care institutions before the new reform, the number of doctors, registered nurses, technicians, and other job positions increased by 25,129, 24,306, 3839, and 34,146 per year on average, respectively, whereas 3288 pharmacists leave their posts every year. Fortunately, there was an instantaneous increase of 1277 pharmacists after the reform (*p* < 0.05). After the new health-care reform, a total number of 54,821 registered nurses (*p* < 0.05) and 2970 pharmacists (*p* < 0.001) chose to devote themselves to the construction of the primary health-care service. However, 28,482 more doctors per year were added compared to the basic increasing trend before 2010, but it was not statistically significant (*p* = 0.066). Additionally, the number of staff in other job positions declined, with an average number of 19,771 a year. (Table 2, Figure 3)

## 4. Discussion

To our knowledge, few studies have directly used data from the *statistical yearbooks* to analyze the changes in primary health-care human resources before and after China’s new health-care reform. We found that the number of primary health workers per 1000 people in China gradually rose from 1.98 in 2003 to 3.07 in 2020, and there was an immediate sharp rise from 2008 to 2009. Subsequently, the number of primary health workers increased at a significant speed, but it still failed to meet people’s basic medical needs. In our study, there was an immediate increase of 51,301 primary health staff from 2009 to 2010, and 6007 more primary health workers per year were added after the reform compared to the basic increasing number before 2010, but both were not statistically significant. The human resources of primary health-care institutions in the eastern region were relatively richer than those in the central and western regions. The average number of staff in community health service centers (stations) after the new reform was 3.24 times than that before the new reform, while people working in village health clinics decreased year by year. The annual increased number of health workers in township health centers was 2.63 times than that before the reform, with a total annual increase of 34,192 people. The growth trend changed the most in western China. From 2003 to 2009, 3288 pharmacists left their posts every year, but fortunately, there was an instantaneous increase of 1277 pharmacists after the reform. Numerous registered nurses and pharmacists were devoted to the construction of the primary health-care service since 2010, while the increase in the number of doctors per year was not statistically significant (*p* = 0.066).

Achieving UHC is a difficult and long-term task that is not unique to China, and many countries are also facing this challenge [6]. It is urgent to strengthen the training of primary health workers due to the insufficient quantity of human resources in primary health-care institutions in China. According to the *Outline of the National Medical and Health Service System Plan (2015–2020)* [11], China should have had at least 3.5 primary health workers for every 1000 permanent residents by 2020. However, the number of primary health workers per 1000 people in 2020 was only 3.07, based on the *Chinese Health Statistics Yearbook 2021*, which indicated a shortage of primary health workers. Our study demonstrated that the difference in the growth trend of primary health-care personnel before and after the new medical reform was not considered to be statistically significant. 

Compared with the circumstance before the reform, there was an apparent instantaneous increase in pharmacists after the intervention. Yearly since 2010, 54,821 registered nurses and 2970 pharmacists have devoted themselves to the construction of the primary health-care service. However, 28,482 more doctors per year were added compared to the basic increasing trend before 2010, but this was not statistically significant. China’s primary health-care personnel team is expanding, driven by the relevant policies. However, it still cannot meet the needs for primary medical and health services for several reasons [5]. In China, general practitioners are the direct providers of core services in primary-level medical and health institutions and the gatekeepers of people’s health [12]. However, there are only about 400,000 primary-level general practitioners in China, with each serving more than 4500 people on average [12]. In the less economically developed regions, such as the central and western regions, the shortages of general practitioners, primary medical workers, and professional and technical personnel in the laboratory and radiology are particularly acute [12]. Due to the lack of professional and technical personnel, much of the medical equipment has been idle for a long time in certain primary health-care institutions [12]. Unsatisfactory salaries, poor working environments, few training opportunities, and imperfect incentive mechanisms may cause the primary medical institutions to suffer serious brain drain [4,8,13,14,15,16].

Due to the specific regional economic development in China, the allocation of health resources was also unbalanced [17]. Although the western region continued to catch up, with the fastest growth rate of health human resources, the eastern region was still significantly richer than the central and western regions in primary health human resources. Therefore, it is essential to attach importance to the construction of primary health personnel teams and increase financial investment in particular regions and sections [17]. In addition, we are supposed to narrow the gap between regions in health manpower by integrating health human resources and access to health services according to geographical, political, economic, and demographic factors [15,18]. With the aging of China’s population and the gradual transformation of the disease spectrum, the training of primary health personnel and the expansion of access to primary health services will become the top priority in the development of medical and health services in the future [4].

After the reform, the growth rate of the number of community health service centers (stations) decreased while the average annual growth rate of the number of health personnel in township health centers increased significantly. Except for the western region, the number of personnel in village clinics in China decreased year by year after the new medical reform. Among them, the decline in village clinics may be because registered nurses were not included in this category before 2007, resulting in an inflated growth rate before the reform. The acceleration of urbanization has greatly reduced the rural population, which may be one of the reasons why the demand for rural medical and health services has decreased [19]. It is key to maintain the development momentum of community health service institutions to focus on primary health services, improve the diagnosis and treatment level, increase people’s trust in them, increase pay and job satisfaction, and reduce the siphon effect of hospitals on primary health talent [4,5,8,13,14,15,16]. The rapid increase in the number of township health personnel is mainly attributed to the continuous expansion of health personnel in western China [18,20]. It, to some extent, reflects the role of policies on medical reform in adjusting the regional distribution of health human resources and promoting the regional coordinated development of primary health-care capacity [20].

There are some limitations to this study. First, the new health-care reform of primary health-care institutions is a comprehensive reform integrating various policies, and its achievements in the human resources of primary medical institutions should be assessed in accordance with the specific background and implementation of each province. Second, as the database we used (*Chinese Health Statistics Yearbook*) did not provide the specific human resource data of different job positions of each province, we were unable to analyze this part by regions. Moreover, due to the large time span of this study, the definition of personnel calculation and classification might be different in some details. To maintain the consistency of classification, the data we finally chose may be smaller in some years, thus underestimating the development of human resources in primary health in China. To avoid the impact of COVID-19, we did not include data in 2020.

## 5. Conclusions

Totally, the primary health-care human resource in China is constantly expanding, especially the development of township health centers and the increase in registered nurses and pharmacists. However, our results demonstrated that the quantity of human resources in primary health-care institutions is still insufficient, especially for doctors, and the problem of unbalanced allocation urgently needs to be solved. We summarized the development of primary health-care institutions in China, driven by new medical reform policies, providing decision support for promoting the rational and effective development of those medical institutions. Thus, with the aging of China’s population and the gradual transformation of the disease spectrum, the training of primary health personnel and the expansion of access to primary health services will become the top priority in the development of medical and health services in the future.

## Figures and Tables

**Table 1:** Changes in the number of staff in primary health-care institutions in China ^a^.

Indicators	2003–2009		2010–2019	
	$N\;(\bar{X})\pm S$	Annual Growth Rate (%)	$N\;(\bar{X})\pm S$	Annual Growth Rate (%)
**Primary Health-Care Institutions ^b^**				
Total	2,770,318 ± 201,403	3.52	3,638,247 ± 274,920	2.67
**Institutions**				
Community health service centers (stations) ^c^				
Eastern ^b^	136,486 ± 29,455	23.13	285,294 ± 32,130	5.05
Central ^b^	57,587 ± 18,714	38.61	120,973 ± 11,355	3.69
Western ^b^	36,169 ± 11,952	39.81	95,346 ± 15,847	7.21
Total ^b^	230,242 ± 60,031	29.25	501,613 ± 59,220	5.12
Total	154,504 ± 82,664	30.38	501,613 ± 59,220	5.12
Township health centers ^d^				
Eastern	381,803 ± 16,252	0.60	455,036 ± 25,641	4.71
Central	383,145 ± 16,119	1.68	428,465 ± 18,915	1.39
Western	272,016 ± 15,344	1.66	396,366 ± 53,790	1.85
Total	1,036,964 ± 46,391	1.27	1,279,868 ± 97,821	2.56
Village clinics ^e^				
Eastern	377,766 ± 29,475	3.82	457,499 ± 10,990	−0.20
Central	370,094 ± 43,381	6.40	490,586 ± 14,905	0.04
Western	299,295 ± 23,935	3.26	371,427 ± 10,991	0.58
Total	1,047,097 ± 91,651	4.54	1,319,511 ± 34,718	0.11
**Job Positions**				
Doctors				
Total	830,763 ± 56,908	3.52%	1,123,547 ± 156,049	4.71%
Registered Nurses				
Total	328,331 ± 59,321	8.25%	659,211 ± 161,096	8.35%
Pharmacists				
Total	122,451 ± 9002	−1.71%	135,384 ± 9237	2.16%
Technicians				
Total	57,641 ± 10,635	6.86%	90,721 ± 11,742	4.00%
Other Job Positions				
Total	1,431,132 ± 85,274	2.81%	1,629,385 ± 65,750	−1.14%

^a^ The classification of regions strictly followed the definition of *statistical yearbook 2020*; ^b^ The number of personnel in primary health-care institutions was obtained directly from *statistical yearbook*, other than the sum of those three institutions; ^c^ Due to the lack of population in community health service centers (stations) in eastern, central, and western China from 2003 to 2006, the labeled classification only contains data from 2007 to 2019, that is, the corresponding table contained the population and average change rates from 2007 to 2019, 2007 to 2009, and 2010 to 2019; ^d^ Due to the loss and confusion of classification of more than half of the data, the personnel data of township health centers in Beijing and Shanghai were deleted; ^e^ To maintain the consistency of data from 2003 to 2019, registered nurses were excluded from the number of village clinics.

**Table 2:** Interrupted time series analysis of changes in the number of personnel in primary health-care institutions in China from 2003 to 2019 ^a^.

Indicators	Before the Health-Care Reform (2003–2009)			Level Change Immediately after the Health-Care Reform (2009–2010)			After the Health-Care Reform (2010–2019)		
	β_1_	95% CI	*p* Value	β_2_	95% CI	*p* Value	β_3_	95% CI	*p* Value
**Primary Health-Care Institutions ^b^**									
Total	91,581	54,273~128,889	<0.001	51,301	−108,368~210,970	0.500	6007	−44,649~56,663	0.802
**Institutions**									
Community health service centers (stations) ^c^									
Total	37,416	31,774~43,058	<0.001	96,717	65,983~127,450	<0.001	−15,032	−21,561~−8502	<0.001
Township health centers ^d^									
Eastern	2665	−2698~8028	0.303	18,199	−6984~43,382	0.143	6256	−760~13,272	0.076
Central	5833	1995~9671	<0.05	−9202	−27,140~8735	0.288	693	−4341~5727	0.771
Western	4594	198~8990	<0.05	17,598	−1786~36,982	0.072	13,996	8088~19,904	<0.001
Total	12,982	−213~26,177	0.053	24,781	−33,476~83,038	0.375	21,210	3485~38,935	<0.05
Village clinics ^e^									
Eastern	12,893	8592~17,194	<0.001	35,218	11,791~58,645	<0.05	−14,461	−19,439~−9484	<0.001
Central	18,377	11,425~25,329	<0.001	51,000	13,129~88,870	<0.05	−19,269	−27,315~−11,223	<0.001
Western	6155	−487~12,796	0.067	45,100	8922~81,277	<0.05	−5619	−13,305~2068	0.138
Total	37,396	20,682~54,109	<0.001	131,491	40,449~222,533	<0.05	−39,320	−58,663~−19,977	<0.001
**Job Positions**									
Doctors									
Total	25,129	3402~46,856	<0.05	−10,084	−86,289~66,121	0.780	28,482	−2149~59,113	0.066
Registered Nurses									
Total	24,306	7778~40,834	<0.05	16,543	−38,889~71,975	0.530	30515	7189~53,841	<0.05
Pharmacists									
Total	−3288	−4982~−1595	<0.05	1277	3499~21,945	<0.05	6258	4299~8218	<0.001
Technicians									
Total	3839	1603~6075	<0.05	495	−8873~9863	0.911	49	−3007~3106	0.973
Other Job Positions									
Total	34,146	19,105~49,186	<0.001	150,641	68,713~232,569	<0.05	−53,917	−71,323~−36,511	<0.001

^a^ The classification of regions strictly followed the definition of *statistical yearbook 2020*; ^b^ The number of personnel in primary health-care institutions was obtained directly from *statistical yearbook*, other than the sum of those three institutions; ^c^ Due to the lack of population in community health service centers (stations) in eastern, central, and western China from 2003 to 2006, we only included national data from 2003 to 2019 in the labeling classification; ^d^ Due to the loss and confusion of classification of more than half of the data, the personnel data of township health centers Beijing and Shanghai were deleted; ^e^ To maintain the consistency of data from 2003 to 2019, registered nurses were excluded from the number of village clinics.

## Data Availability

All data in the study are available from public databases by request. The data can be found at https://data.cnki.net/yearbook/Single/N2022010155 (accessed on 6 March 2022).
